# Everyday Ageism: Perspectives from People Aged 85+ Using Participatory PhotoVoice Methodology

**DOI:** 10.1093/geroni/igaf122.2201

**Published:** 2025-12-31

**Authors:** Sophia Ashebir, Taylor Brennan, Niels Wu, Lisa D’Ambrosio

**Affiliations:** Massachusetts Institute of Technology, Cambridge, Massachusetts, United States; MIT, Boston College, Cambridge, Massachusetts, United States; Massachusetts Institute of Technology, Cambridge, Massachusetts, United States; Massachusetts Institute of Technology, Cambridge, Massachusetts, United States

## Abstract

While research on ageism is increasingly well-documented, little attention has been paid to how people may personally experience ageism during specific age periods in their older adulthood. Methods leveraging approaches from participatory action research to explore how the oldest of older adults (those aged 85+) experience ageism are even more scarce. This study addresses this gap by employing mixed methods, including PhotoVoice, a qualitative method leveraging participant-taken photographs, and virtual semi-structured interviews, to investigate ageism through the perspectives of a sample of adults aged 85+. Participants (N = 30) were recruited from the MIT AgeLab 85+ Lifestyle Leaders panel, a longitudinal study of U.S. octogenarians and nonagenarians, the MIT AgeLab Volunteer Database, and snowball sampling. Using an inductive, team-based thematic analysis approach, we identified a spectrum of experiences related to ageism, including covert and overt manifestations, positive and negative encounters, and external versus internalized perceptions. Participants highlighted challenges related to ageism in specific domains such as product design, healthcare, and the built environment. Additionally, instances of positive ageism—where aging was met with preferential treatment or social advantage—were particularly salient in this sample. The use of PhotoVoice as a visual storytelling tool offered a richer, multidimensional understanding of experienced ageism, further supplementing findings from interviews. Findings underscored the importance of co-creating knowledge with older adults and exploring alternative methodologies to understand lived experiences. The study contributes to both theoretical and applied discussions on ageism, emphasizing the value of inclusive, participatory research.

